# Exposure to pornographic material and perpetration of intimate partner violence among young men in Mwanza Tanzania

**DOI:** 10.1007/s10389-024-02262-7

**Published:** 2024-04-17

**Authors:** Diana Aloyce, Heidi Stöckl, Neema Mosha, Donati Malibwa, Simon Sichalwe, Ramadhan Hashim, Philip Ayieko, Saidi Kapiga, Gerry Mshana

**Affiliations:** 1 Mwanza Intervention Trials Unit, Isamilo Street, Ilemela Mwanza, Tanzania; 2 Institute for Medical Information Processing, Biometry, and Epidemiology, Faculty of Medicine, Ludwig-Maximilians-Universität Munich, Elisabeth-Winterhalter-Weg 6, 81337 Munich, Germany; 3 London School of Hygiene and Tropical Medicine, Keppel Street, London, WC1E 7HT UK; 4 National Institute for Medical Research, Isamilo Street, Ilemela Mwanza, Tanzania; 5 Pettenkofer School of Public Health, Munich, Germany

**Keywords:** Pornography, Intimate partner violence perpetration, Young men, Negative Masculinity, Africa

## Abstract

**Aim:**

Intimate partner violence (IPV) is a public health concern that negatively impacts women’s health. Preliminary evidence from high-income countries suggests that IPV is linked with exposure to pornographic materials among men, by encouraging negative norms of masculinity. To generate evidence from low and middle-income countries, we examined the relationship between frequent exposure to pornography and IPV perpetration among young men in Mwanza, Tanzania.

**Subject and methods:**

We conducted a cross-sectional survey among 1002 randomly selected young men aged 18 to 24 living in Mwanza, Tanzania.

**Results:**

Of the 828 ever-partnered young men included in the study, 396 (47.8%) reported viewing pornography in the past 12 months, with 14.1% viewing it at least once a week. In the last 12 months, 21.4% of participants reported perpetrating sexual IPV, while 43.2% and 15% reported to have perpetrated emotional and physical IPV respectively. After adjusting for covariates, exposure to pornography was significantly associated with sexual (aOR = 2.77 95% CI 1.51 – 5.08), emotional (aOR = 1.84 95% CI: 1.01 – 3.37) and physical (aOR = 1.65 95% CI 1.00 – 2.74) IPV perpetration.

**Conclusion:**

Frequent exposure to pornography was associated with sexual, emotional, and physical IPV perpetration. Interventions to prevent violence against women therefore need to address men’s exposure to pornography.

## Introduction

Men’s perpetration of intimate partner violence (IPV) is a serious public health problem that leads to adverse physical and mental health problems in women (García-Moreno and Stöckl 2009). The number of women experiencing IPV has remained high over many decades (Abbafati et al. 2020; WHO 2021). Globally, one in four women experience physical and/or sexual IPV during their lifetime (Sardinha et al. 2022). Women aged 15–49 years in sub-Saharan Africa are reported to experience among the highest rates of physical and/or sexual IPV, ranging between 27%-44% in a lifetime and 14%-32% in the past year (Sardinha et al. 2022). In Tanzania, more than 40% of women report to have experienced IPV perpetrated by men in their lifetime (Kapiga et al. 2017; Tanzania Ministry of Health and Social Welfare 2016).

Emerging evidence from high income countries suggests a relationship between exposure to pornography and perpetration of IPV among men (Brem et al. 2021; Jongsma and Fritz 2022; Mulumeoderhwa and Harris 2015; Spadine et al. 2022). Pornography captures nude photographs, sex tapes, movies or films, erotic novels, magazines or writings depicting sexual activity or erotic behaviours designed to arouse sexual excitement (Garner 2014; Jongsma and Fritz 2022). Due to the advancement of information technology and the growth of internet use, pornographic materials are widely available globally (Alexy et al. 2009; Bridges et al. 2010). Evidence also suggests that young men frequently consume pornographic materials (Bridges et al. 2010; Lim et al. 2017; Mulumeoderhwa and Harris 2015; Sun et al. 2016). A study in the United States among male college students aged between 18 and 29 reported that 85.2% of them were frequently viewed pornography and among those 13.5% used it on a daily basis for masturbation purposes (Sun et al. 2016). Another study in Australia conducted among male and female participants aged between 15 to 29 years reported a higher exposure to pornographic materials among young men than among women. In that study, 99% of male participants were viewed pornography in the past 12 months, 46% were exposed on weekly basis and 35% on daily basis (Lim et al. 2017).

Research has documented several negative impacts of pornography on men, including fostering sexual aggression, increasing problems with sexual arousal, lower relationship commitment, less fidelity and creating expectations of sexual acts in pornographic scenes to be replicated in their real sexual lives (Alexy et al. 2009; Bridges et al. 2010; Jongsma and Fritz 2022; Maitse 1998; Mulumeoderhwa and Harris 2015; Tarzia and Tyler 2021). Furthermore, pornography portrays negative and hostile masculinity (Baer et al. 2015), a major driving force for men’s perpetration of IPV against women (Connell and Messerschmitt 2005; Sikweyiya et al. 2020). The majority of pornographic materials tend to denigrate women and play with violent or dominating fantasies. A content analysis of the best-selling pornographic videos reports 88.2% of it to contain physical violent acts against women, 48.7% to contain verbal aggression and with women acting with pleasure or neutrality in 95% of the time when these acts are committed (Bridges et al. 2010). Hence there are serious concerns regarding the consumption of pornography given the influence it exerts on men’s ideas about sexual intercourse, and it’s potential to trigger violence against women.

There is a lack of research studies examining the relationship between exposure to pornography and IPV perpetration in sub-Saharan Africa. The few studies that exist have acknowledged the association between pornography and rape or physical violence among partners and non-partners (Bekele et al. 2011; Maitse 1998; Mulumeoderhwa and Harris 2015). However, they have explored this information with female samples only, except for one study from the Democratic Republic of Congo that used a sample of male and female high school students. The study found that exposure to pornography was associated with forced sex, rape and sexual exploitation against female high school students (Mulumeoderhwa and Harris 2015). Our study therefore aims to explore the relationship between exposure to pornography and the perpetration of different forms of IPV in Mwanza, Tanzania.

## Methods

### Study design and setting

This study is a cross-sectional survey, conducted between June 2021 and March 2022, among a representative sample of young men in Ilemela and Nyamagana districts of Mwanza city, Tanzania. Mwanza is the second largest city in Tanzania located on the southern shores of Lake Victoria. It is a cosmopolitan city, and due to its strategic location supporting diverse economic activities, it is inhabited by a wide range of people from across the country.

### Study sampling procedures and participants recruitment

Local administrative units and households within each of the two districts in Mwanza (Ilemela and Nyamagana) were sampled using a probability-based multi-stage cluster sampling approach. At first, a sampling frame comprising a list of all 37 wards in the two districts was prepared. Thereafter, 13 wards that were already participating in another similar study among women were excluded from the list. The remaining 24 wards were stratified into two groups, densely and sparely populated wards and then three wards were randomly selected from each group with probability proportional to size. Finally, a random sample of four streets were selected from each of the six wards making a total of 24 streets.

Field workers visited all selected streets and marked the boundaries using Global Positioning System (GPS) coordinates. Subsequently, 120 GPS coordinates were randomly generated within each street using Q-GIS software. The coordinates were ordered randomly and then visited by the field team in that order. At each point, field workers visited the two houses nearest to the coordinates. In each household they prepared a list of potential eligible participants aged between 18 and 24 years residing in the house. From the list, one participant was randomly selected in each household and interviewed after providing informed consent. The team then moved to the next coordinate on the list. A total of 1002 men aged 18 to 24 years were recruited into the survey. Data was collected using face-to-face interviews. Due to sensitivity of the IPV questions (Barros and Schraiber 2017), participants were handed tablets to self-complete these questions with the help of audio recordings and colour-coded responses.

### Measures

The study assessed perpetration of three forms of IPV as outcome variables. These included perpetration of sexual, emotional and physical IPV. The three forms of IPV were selected because they constitute acts such as forced sex, use of weapons and verbal insults and others that are commonly reported in pornographic materials (Bridges et al. 2010; Sun et al. 2016). These forms of IPV were measured using an index score of 3 acts of sexual IPV, 6 acts of emotional abuse and 3 acts of physical IPV adapted from the Intervention with Microfinance for AIDS and Gender Equity (IMAGE) (Fulu et al. 2013) and the Sonke Change Trial questionnaire (Christofides et al. 2020). See Table 1 for an overview of the individual IPV questions included. A binary variable for each of those forms of IPV was generated from a “yes” response to any of the IPV perpetration questions that was considered as perpetrating that specific form of violence.

**Table 1:** Survey questions on intimate partner violence perpetration

**Physical Intimate Partner Violence**
Thinking of any partner you ever had in your whole life, have you ever slapped, pushed or shoved her or thrown something at a partner that could hurt her?
Thinking of any partner you ever had in your whole life, have you ever hit a partner with a fist or something else that could hurt her, kicked, dragged, beaten, choked or burned her?
Thinking of any partner you ever had in your whole life, have you ever threatened to use or actually used a gun, knife or other weapon against a partner?
**Sexual Intimate Partner Violence**
Thinking of any partner you ever had in your whole life, have you ever forced a partner to have sex with you when she did not want to?
Thinking of any partner you ever had in your whole life, have you ever had sex with a partner when you knew she didn’t want it, but you believed she should agree because she was your wife/partner?
Thinking of any partner you ever had in your whole life, have you ever forced a partner to do something sexual that she did not want to?
**Emotional Intimate Partner Violence**
Thinking of any partner you ever had in your whole life, have you ever spread rumours about her or tried to turn her friends against her?
Thinking of any partner you ever had in your whole life, have you ever spoken to her in a mean (hostile) tone of voice?
Thinking of any partner you ever had in your whole life, have you ever insulted her or deliberately said things to make her feel bad about herself?
Thinking of any partner you ever had in your whole life, have you ever made fun of her, belittled her or humiliated her in front of other people?
Thinking of any partner you ever had in your whole life, have you ever tried to scare or intimidate her on purpose, for example by the way you looked at her, by shouting, or by smashing things?
Thinking of any partner you ever had in your whole life, have you ever threatened to hurt or actually hurt people she cares about as a way of hurting her, or damage things of importance to her?

The main exposure for the study was participant’s exposure to pornography adapted from the NFSS Survey instrument (Regnerus et al. 2016). The question asked how often participants watched pornographic materials such as internet sites, magazines or movies in the past year. A categorical variable was generated with a “never” response for never watching pornographic materials, “less frequent” for watching pornography once to three times a month and “frequently” for watching pornography at least once a week.

Other variables that were assessed in the questionnaire and could potentially have a confounding effect in the relationship between IPV perpetration and pornography were identified. These included demographics such as education that was re-coded as a categorical variable with 3 categories 1) no/incomplete primary education, 2) incomplete/complete primary education / and 3) secondary and above. Age was re-coded as a categorical variable with categories of age below 20 and age above 20. Also, employment was coded as a binary variable with a yes or no response.

Other covariates that were assessed in the study were depression, alcohol use as well as tobacco and/or substance use. Depression was assessed using 9 questions from the Patient Health Questionaire-9 (PHQ-9), a validated depression screening tool (Fawzi et al. 2020). The total scores for the tool range from 0 to 27. A score of 0–4 was categorized as none or minimal depression, 5–9 mild depression, 10–14 moderate depression, 15–19 moderately severe, and 20–27 severe depression. The variable was then re-coded into a binary variable combining the mild, moderate, moderately severe and severe depression into one group and none or minimal depression as the second group. Alcohol use was assessed using 10 questions of the WHO Alcohol Use Disorder Identification Test (Babor et al. 2001). A maximum score of 40 was expected whereby 0 stands for abstainers, 1 to 7 for low-risk alcohol consumption, 8 to 14 for harmful alcohol consumption and of 15 and above alcohol dependence. The current use of tobacco and substance use was assessed using questions from the Demographic and Health Survey of 2015 (Tanzania Ministry of Health and Social Welfare 2016). A single variable for tobacco and substance use was created from a “yes” response to the questions assessing either of the two.

### Statistical analysis

Data analysis was conducted using STATA version 15.0 (StataCorp, TX, USA). Analysis was restricted to young men who were currently or had ever been in an intimate relationship (n = 828). We initially determined the frequency and percentages of demographic characteristics, reported watching of pornographic information and each form of IPV. Bivariate and multivariable logistic regression models were used to determine the associations between different forms of IPV perpetration and exposure to pornography. In both the bivariate and multivariable models, we adjusted for the street-level clustering effect by using robust standard error. All variables with p-value less than 0.05 in bivariate analysis were identified and included in multivariable models. All variables with a p-value of less than 0.05 were retained in the final multivariable models. We used odds ratios with their corresponding 95% confidence interval to summarise the strength of the associations between different forms of IPV with pornography.

### Ethical consideration

The study was approved by the Tanzanian National Health Research Ethics Committee, (reference: NIMR/HQ/R.8a/Vol.IX/2991), from the ethics committee of the medical faculty of the Ludwig-Maximilians-University, Germany (reference: 21–0508) and the London School of Hygiene and Tropic Medicine, United Kingdom (reference: 16,121). All study participants provided informed consent after receiving information about the study objectives and procedures. Interviews were conducted in private locations of participants’ choice by specially trained male interviewers. Referral information was provided to all participants at the end of the interviews, but none of the men wanted to be referred.

## Results

The median age of the 828 ever-partnered participants was 21 (interquartile range: 19–23) years. More than a half of the participants had attained their secondary education or above and 606(73.2%) worked either as skilled or unskilled laborer during the past 12 months. Among all participants, 195(23.6%) reported to have drunken alcohol during the past 12 months, 57(6.9%) used tobacco or substance in the past 12 months and 333(40.2%) screened positive for either mild or severe depression. See Table 2.

**Table 2:** Background characteristics of the study participants (N = 828)

Demographic Characteristics	N = 828	(%)
Districts		
Ilemela	369	44.6
Nyamagana	459	55.4
Age group		
18–20	339	40.9
21–24	489	59.1
Level of Education		
No/incomplete primary education	96	11.6
Primary education and incomplete secondary	315	38.0
Secondary and above	417	50.4
Employment Status (in the past 12 months)		
Employed	606	73.2
Not employed	222	26.8
Other Covariates		
Alcohol consumption (in the past 12 months)		
Low-risk consumers	102	12.3
Harmful consumers	66	8.0
Dependent consumers	27	3.3
Abstainer	633	76.4
Tobacco & Substance use (in the past 12 months)		
Yes	57	6.9
No	771	93.1
Depression		
Yes	333	40.2
No	495	59.8

### Descriptive statistics and bivariate correlations of the outcomes and main covariate

Perpetration of all forms of IPV was high in our sample, with 222(26.8%) of participants perpetrating sexual IPV in their lifetime and 177(21.4%) in the past 12 months, 423(51.1%) perpetrating lifetime emotional IPV and 358(43.24%) reporting it in the past 12 months and 177(21.4%) perpetrating lifetime physical IPV and 124(15.0%) reporting it in the past 12 months. In the last 12 months, 396 (47.8%) participants reported viewing pornographic material, 14.1% frequently.

In Table 3 we present the prevalence of different forms of IPV perpetration in the past 12 months by frequency of exposure to pornography. Overall, the prevalence of perpetration of sexual and emotional IPV increased with higher frequency of viewing pornography. Although the prevalence of perpetration of physical IPV was highest among those reporting frequently viewing pornography, we observed the same prevalence of IPV perpetration among those not exposed to pornography and those reporting less frequent exposure to pornography.

**Table 3:** The prevalence of IPV by reported exposure to pornography among young men in Mwanza, Tanzania (N = 828)

Pornography	n (%)	Sexual IPV		Physical IPV		Emotional IPV	
		Perpetration		Perpetration		Perpetration	
		YES %	95%CI	YES (%)	95%CI	YES (%)	95%CI
Never	432 (52.2)	17.8	14.3 – 22.8	14.4	11.2—18.0	36.8	32.3 – 41.6
Less frequent	340 (41.0)	22.7	18.3 – 27.5	14.4	10.9 – 18.6	49.4	44.0 – 54.9
Frequent	56 (6.8)	41.1	28.1 – 55.0	23.2	13.0—36.4	55.4	41.5 – 68.7

### Bivariate and multivariable analysis

In the unadjusted models, frequent exposure to pornography was associated with higher odds of sexual IPV perpetration (OR = 3.21 95% CI: 1.83 – 5.64, p < 0.001) and physical IPV perpetration (OR = 1.8 95% CI: 1.06 – 3.06, p = 0.028) (see Table 4). Emotional IPV was significantly associated with both frequent exposure to pornography (OR = 2.13 95% CI: 1.24 – 3.66, p = 0.006) and infrequent exposure (OR: 1.68 95% CI: 1.21- 2.33, p = 0.002) as compared to never being exposed to this information.

**Table 4:** Bivariate association of perpetrating different forms of IPV with pornography and other factors (N = 828)

Variable	n(%)	Sexual IPV Perpetration		Physical IPV Perpetration		Emotional IPV Perpetration	
		OR	95% CI	OR	95%CI	OR	95%CI
Pornography							
No	432 (52.2)	1		1		1	
Less frequently	340 (41.0)	1.35	0.91- 1.10	1.0	0.66 – 1.54	1.68	1.21 – 2.33
Frequently	56 (6.8)	3.21	1.83 – 5.64	1.8	1.06—3.06	2.13	1.24 – 3.66
Employment							
No	222 (26.8)	1		1		1	
Yes	606 (73.2)	1.7	1.08 – 2.27	1.6	0.98 – 2.72	1.3	0.96 – 1.81
Age group							
Below 20 years	339 (40.9)	1		1		1	
Above 20 years	489 (59.1)	1.5	1.31 – 2.22	0.9	0.61 – 1.19	1.4	0.97 – 1.87
Education							
No & Incomplete primary	96 (11.6)	1		1		1	
Primary &Incomplete		1.1	0.97 – 2.25	0.6	0.38 – 1.04	1.2	0.82 – 1.81
secondary	315 (38.0)						
Sec education & above	415 (50.4)	1.0	1.45 – 3.29	0.4	0.25 – 0.72	1.4	0.84 – 2.27
Depression							
No	495 (59.8)	1		1		1	
Yes	333 (40.2)	2.7	1.27 – 2.28	1.5	0.89 – 2.46	2.1	1.61 – 2.73
Alcohol use (Based on AUDIT scale*)							
Abstainers	633 (76.4)	1		1		1	
Low risk	102 (12.3)	1.3	0.77 – 2.21	1.2	0.51 – 2.67	1.2	0.76 – 1.93
Harmful	66 (8.0)	3.1	1.59 – 5.89	4.5	2.26 – 8.83	4.1	2.57 – 6.49
Dependent	27 (3.3)	3.4	1.15 – 9.96	3.7	1.57 – 8.57	1.9	0.87 – 4.20
Tobacco/Substance use							
No	771 (93.1)	1		1		1	
Yes	57 (6.9)	2.1	0.71 -5.95	2.6	1.34 – 5.19	2.4	1.37 – 4.16

Perpetration of sexual IPV was also significantly associated with being employed (OR = 1.7 95%CI: 1.08 – 2.27, P = 0.006), depression (OR = 2.7 95%CI: 1.27 – 2.28, p < 0.001) as well as harmful (OR = 3.1 95%CI: 1.59 – 5.89, p = 0.001) and dependent (OR = 3.4 95%CI: 1.15 – 9.96, p = 0.027) alcohol consumption. Physical IPV perpetration was also significantly associated with harmful (OR = 4.5 95%CI: 2.26 – 8.83, p < 0.001) and dependent (OR = 3.7 95%CI: 1.57 – 8.57 p = 0.003) alcohol use and tobacco and or substance use (OR = 2.6, 95%CI: 1.34 – 5.19, p = 0.005). Emotional IPV perpetration was significantly associated with being depressed (OR = 2.1 95%CI: 1.61 – 2.73, p = 0.002), harmful alcohol consumption (OR = 4.1 95%CI: 2.57 – 6.49, p < 0.001), and tobacco and or substance use (OR = 2.4 95%CI: 1.37 – 4.16, p = 0.002).

After adjusting for factors significantly associated with the different forms of IPV in the unadjusted models, all forms of IPV remained significantly associated with frequent exposure to pornography: sexual IPV perpetration (aOR = 2.77 95% CI 1.51 – 5.08, p = 0.001), physical IPV perpetration (aOR = 1.65 95% CI 1.00 – 2.74, p = 0.05) and emotional IPV perpetration (aOR = 1.84 95% CI: 1.01 – 3.37, p = 0.047). Infrequent exposure to pornography was also significantly associated with emotional IPV perpetration (aOR = 1.56 95% CI 1.14 – 2.15, p = 0.006).

## Discussion

To the best of our knowledge, this is the first study to establish consistent associations between frequent exposure to pornography and perpetration of various forms of IPV among a representative sample of young men from sub-Saharan Africa. The findings show a positive association between sexual, physical and emotional IPV perpetration with frequent exposure to pornography. Infrequent exposure to pornographic materials was also associated with emotional IPV perpetration. In addition, other factors such as being depressed, harmful alcohol consumption, and tobacco and substance use were significantly associated with different forms of IPV perpetration in this study.

These findings are similar to other studies that have examined the relationship between frequent exposure to pornography with sexual, emotional and physical IPV perpetration among adult and young men globally and from Africa (Brem et al. 2021; Bridges et al. 2010; Jongsma and Fritz 2022; Mulumeoderhwa and Harris 2015; Sun et al. 2016). Studies that have analyzed the contents of pornographic materials further reported that pornography consumption influences men’s violent sexual behaviors as it exposes men to sexual scripts that frequently portray violence against women as part of sexual pleasure (Bridges et al. 2010; Sun et al. 2016). A longitudinal study in the United States showed that frequent exposure to pornography among men resulted in increased IPV perpetration over time (Jongsma and Fritz 2022). In addition, men who are frequently exposed to pornography were reported to use it for masturbation and expressed diminished enjoyment in the enactment of sexually intimate behaviours with their intimate partners (Sun et al. 2016). This could reduce real-life sexual interest and increase violence perpetration against female partners. These findings lay a foundation for further studies on this relationship to clearly explore and document the mechanisms and pathways through which frequent exposure to pornography among men leads to IPV perpetration.

On the other hand, research has highlighted the widened accessibility of pornographic materials globally through the internet and media devices like mobile phones, computers, gaming systems and others for over a decade now (Alexy et al. 2009). In recent years, the improvement of information technology and the growth of social media platforms widely used by young generations suggest pornographic materials can easily be accessed and shared globally hence increasing the frequency with which they view pornography. Unfortunately, research shows that viewing pornography is addictive especially when the viewers are exposed to many episodes (Whelan and Brown 2021).

Research evidence highlights that around puberty and young age, sex hormones impel young people to seek out sexual experiences and that it is easier for them to obtain it from the internet than in real life (Sharpe and Mead 2021). Pornography is one of the materials that young people are most likely to consume, for sexual education and assistance with sexual arousal (Alexy et al. 2009; Sharpe and Mead 2021; Sun et al. 2016). Sex education is not common in Tanzania (Rosser et al. 2022)**.** As young men seem to receive less real-life sexual education than girls, this might mean that they rely more heavily on peers and the media for information (Sun et al. 2016). In sub-Saharan African countries like Tanzania where the majority of young women get married at a young age (Rosser et al. 2022), some social norms could glorify their need to learn how to satisfy men sexually as one of their main roles in marriage (Mchome et al. 2020). This may put them at an advantageous position to receive sexual education based on real-life experiences at families and community levels compared to men. In this context, men might need to rely more heavily on pornography to receive this information, as they receive less structured initiation.

As pornography consumption may shape men’s sexual behaviours, attitudes and expectations during their sexual encounters with women (Sun et al. 2016), viewers are likely to copy pornographic content including verbal insults, physically aggressive acts such as hitting, spanking and slapping (Bridges et al. 2010) as well as sexually violent behaviours such as non-consensual sex and hostile sex (Maitse 1998). Studies document that some men find the sexual and physically aggressive acts against women in pornography erotic and desirable (Bridges et al. 2010; Maitse 1998; Sun et al. 2016)**.** In addition, women in pornography scenes are widely depicted to be submissive and accept to be sexually objectified by their pornography sexual partners (Bridges et al. 2010; Sun et al. 2016). Young men who get most of their sexual knowledge and sexual arousal from pornography are likely to consider this the normal sexual practice and hence expect this from their real-life partners.

Technology is likely to play a major role in reproducing negative masculinities through exposure to pornographic material and hence a risk factor for IPV perpetrated against women. For example, a recent study from Tanzania highlights the role of mobile phones and communication technologies in reproducing entrenched negative masculinities and gender norms in intimate relationships and other social interactions (Mshana et al. 2022). The role these negative masculinities play in influencing IPV against women, especially in patriarchal societies is well documented (Connell and Messerschmitt 2005; Jewkes et al. 2015; Sikweyiya et al. 2020). As suggested by Mshana et al (2022), our study further stresses the urgent need for researchers to intensively assess the impact of technological advancement in influencing IPV perpetrated against women, especially among the young generation who frequently use these technologies. This will create a good foundation to design evidence-based interventions in reducing IPV, especially among youth in this world of globalization and technology advancement.

Tanzania like many other sub-Saharan countries treats sexuality as an integral matter that needs to be conducted in privacy (Magalla 2022; Rosser et al. 2022). However, due to globalization and spread of information and communication technology materials related to sexuality including pornography are made widely accessible. Following to this, Tanzania has made different initiatives to prohibit and condemn any acts related to pornography by passing and putting into implementation different acts and strong regulations (Magalla 2022; URT 2015). To mention a few, these include the National Information and Communications Technologies Policy of 2003 & 2016, The Tanzania Communication and Regulatory Authority Act of 2003, The Tanzania Electronic and Postal Communications Act, of 2010, the Cyber Crime Act, No.14 of 2015, the Media Service Act, No.12 of 2016, and The Electronic and Postal Communications Online Content Regulations, 2020. Through all these regulations, publishing and spreading pornographic materials in any manner or form is strictly prohibited (Magalla 2022; URT 2015). However, evidence from other countries suggest legal control does not effectively prevent pornography spreading and viewing since it is mostly spread through the internet which respects no boundaries (Alexy et al. 2009; Sharpe and Mead 2021; Sun et al. 2016).

To limit the wide accessibility of pornography in this era of technological advancement high taxation, tariffs and restriction programming codes may be employed worldwide. By joining forces with other nations and different social media platforms these efforts can be successful, especially if they include actions to punish the publishers. On the other hand, comprehensive and friendly sexual education should be well incorporated into school curriculums, and in gender-based community programs towards both young men and women. These should educate both girls and boys on sex based on real-life experiences but also provide men with skills to critically assess and manage the pornography materials. Parents should be included in these programs, especially in sub-Saharan African countries like Tanzania where some societies are documented to have taboos (Rosser et al. 2022) that discourage parents from discussing sex and providing sex education to their children. In addition, the gender transformative programming that is reported to be successful in addressing issues of intimate partner violence among couples (Kyegombe et al. 2021) may be updated and incorporated with knowledge on the negative influences of pornography and addressed among young couples.

Furthermore, with evidence from this and other studies pointing at other risky behaviors such as harmful alcohol consumption and tobacco and substance use to being associated with young men’s perpetration of IPV against their female partners, there is a need to intervene against these risky behaviors altogether. Behavioural change interventions that integrate addressing all these risk behaviours and mental health issues like depression among young men at once can be effective in reducing IPV and these other risky behaviors. For efficiency and sustainability, these behavioural change interventions should start targeting men at an adolescent stage and include creative and user-friendly activities to keep them engaged and interested for a long period of time.

The limitation of this study is that it was not initially designed to assess the relationship between IPV perpetration and frequently watching pornography among young men. It would be necessary for future studies to have a more nuanced measure for pornography use, capturing the duration spent watching pornography, the content watched in the episodes to assess the degree of intimacy as well as whether violence portrayed in those pornographic materials (Bőthe et al. 2020). In addition to this, the cross-section nature of this study does not allow for measuring the casualty of the relationship between exposure to pornography and IPV perpetration among men. Furthermore, the study is focused on young men and does not represent the experiences of adolescent men or men above the age of 25, who may have very different patterns of consuming or accessing pornographic material. The strength of this study is that it is the first from sub-Saharan Africa that utilizes a representative sample of young men to examine the relationship between frequent exposure to pornography and different forms of IPV perpetration against women.

In conclusion, our study shows that frequent exposure to pornography is positively associated with men’s perpetration of physical, sexual and emotional IPV against women. Hence, efforts aiming at reducing IPV perpetration should also invest on reducing and restricting exposure to pornography among young men.

## Data Availability

Participants in the current study have not given approval to share their anonymized data beyond the original authors. To access the data, please contact the authors for joint analysis. Alternatively, for approval to access the data for this study without participation of the original authors, please write to the Medical Research Coordinating Committee, National Institute for Medical research, P.O. Box 6953, Dar es Salaam, Tanzania, referencing IRB Approval No. NIMR/HQ/R.8c/Vol.I/614 and NIMR/HQ/R.8c/Vol. I/837.

## Declarations

### Informed consents

Participation in this study was voluntary and informed consent was obtained from all participants included in the study.
